# Automation of Wilms’ tumor segmentation by artificial intelligence

**DOI:** 10.1186/s40644-024-00729-0

**Published:** 2024-07-02

**Authors:** Olivier Hild, Pierre Berriet, Jérémie Nallet, Lorédane Salvi, Marion Lenoir, Julien Henriet, Jean-Philippe Thiran, Frédéric Auber, Yann Chaussy

**Affiliations:** 1 Department of Pediatric Surgery, CHU Besançon, 3 boulevard Fleming, Besançon, F-25000 France; 2 Université de Franche-Comté, FEMTO-ST Institute, DISC, Besançon, F-25000 France; 3 Department of Radiology, CHU Besançon, Besançon, F-25000 France; 4 Signal Processing Laboratory 5 (LTS5), Ecole Polytechnique Fédérale de Lausanne (EPFL), Lausanne, 1015 Switzerland; 5 University Hospital Center (CHUV) and University of Lausanne (UNIL), Lausanne, 1011 Switzerland; 6 Université de Franche-Comté, SINERGIES, Besançon, F-25000 France

**Keywords:** Artificial intelligence, Deep learning, Segmentation, 3D reconstruction, Wilms’ tumor

## Abstract

**Background:**

3D reconstruction of Wilms’ tumor provides several advantages but are not systematically performed because manual segmentation is extremely time-consuming. The objective of our study was to develop an artificial intelligence tool to automate the segmentation of tumors and kidneys in children.

**Methods:**

A manual segmentation was carried out by two experts on 14 CT scans. Then, the segmentation of Wilms’ tumor and neoplastic kidney was automatically performed using the CNN U-Net and the same CNN U-Net trained according to the OV^2^ASSION method. The time saving for the expert was estimated depending on the number of sections automatically segmented.

**Results:**

When segmentations were performed manually by two experts, the inter-individual variability resulted in a Dice index of 0.95 for tumor and 0.87 for kidney. Fully automatic segmentation with the CNN U-Net yielded a poor Dice index of 0.69 for Wilms’ tumor and 0.27 for kidney. With the OV^2^ASSION method, the Dice index varied depending on the number of manually segmented sections. For the segmentation of the Wilms’ tumor and neoplastic kidney, it varied respectively from 0.97 to 0.94 for a gap of 1 (2 out of 3 sections performed manually) to 0.94 and 0.86 for a gap of 10 (1 section out of 6 performed manually).

**Conclusion:**

Fully automated segmentation remains a challenge in the field of medical image processing. Although it is possible to use already developed neural networks, such as U-Net, we found that the results obtained were not satisfactory for segmentation of neoplastic kidneys or Wilms’ tumors in children. We developed an innovative CNN U-Net training method that makes it possible to segment the kidney and its tumor with the same precision as an expert while reducing their intervention time by 80%.

## Backgroung

Wilms’ tumor, or nephroblastoma, is one of the most common malignant tumors in children, affecting 1 in 10,000 children [1, 2]. Irrespective of the therapeutic protocol followed (International Society of Paediatric Oncology or Children’s Oncology Group), surgery retains an essential role in the care of such children. 3D reconstruction of the neoplastic kidney, based on patient imaging data, provides several advantages: pre-operative surgical planning, anticipation of operative risks (especially vascular), help in the selection of patients who can benefit from nephron-sparing surgery, and improvement of the information provided to families [3]. Currently, these 3D reconstructions are not systematically performed in clinical practice. Indeed, the construction of 3D models requires a preliminary segmentation phase (assigning a label to each pixel of the image), which can be extremely time-consuming and a source of human error when performed manually [3, 4].

Artificial intelligence (AI) encompasses a set of concepts and technologies that enable machines to simulate human intelligence. It uses artificial neural networks, mathematical logic, and computer science. Thus, AI can be defined as a set of algorithms that gives machines the ability to reason or perform certain cognitive functions such as problem-solving, object or word recognition, and decision-making [5]. Deep Learning refers to a subset of machine learning techniques that involve training and using artificial neural networks with multiple layers to learn and extract complex patterns and representations from data. There are different kinds of deep learning architectures that can be used for medical image analysis, and most of them are built from convolutional neural networks (CNN) [4]. Thus, CNN are a type of deep learning model specifically designed for processing and analyzing structured data, such as images or time-series data. They utilize convolutional layers to automatically extract hierarchical features from the input data, making them highly effective for tasks like image recognition, classification or segmentation [6]. In the context of tumor pathology, these tools can be used for tumor segmentation, differential diagnosis, tumor staging and grading [4].

The objective of our study was to develop an artificial intelligence tool to, as much as possible, automate the segmentation of the neoplastic kidney in order to limit the need for intervention by a human expert and thus allow it to be performed in routine clinical practice.

## Materials and methods

We built a database from 14 CT scans of 12 patients who had been treated for Wilms’ tumor in our department at the University Hospital of Besançon, France. All scanners had an arterial contrast phase, and 5 scanners had a late acquisition time allowing the urinary tract to be assessed. For each CT scan, we performed manual or semi-automatic segmentation of healthy kidneys, neoplastic kidneys (preserved renal parenchyma around the tumor), Wilms’ tumors, arterial vascularization, venous vascularization, and urinary cavities, using 3D Slicer software version 4.8.1 (https://www.slicer.org/). This manual segmentation was carried out by two different experts in order to calculate the inter-individual variability (one pediatric surgeon and one pediatric radiologist with more than 5 years’ experience in pediatric oncology). The patient demographics and segmentation method were described in a previous paper [3]. These established data were then available for the development and training of AI tools.

### Convolutional neural networks (CNN)

Segmentation of the neoplastic kidney and the tumor was initially performed with U-Net [7], which is the most commonly used CNN for medical image segmentation. U-Net has two stages: a down-sampling stage (an encoder process uses the max-pooling strategy to compress image features) and an up-sampling stage (a decoder process uses the unpooling strategy to outpout the results) [8]. For each patient p, the U-Net has been trained over all others the patients except p, during 200 epochs (the number of epochs is a hyperparameter of CNN that controls the number of complete passes through the training dataset), with a batch size of 16 (the batch size is a hyperparameter of CNN that controls the number of training samples to work through before the model’s internal parameters are updated).

**Fig. 1:**
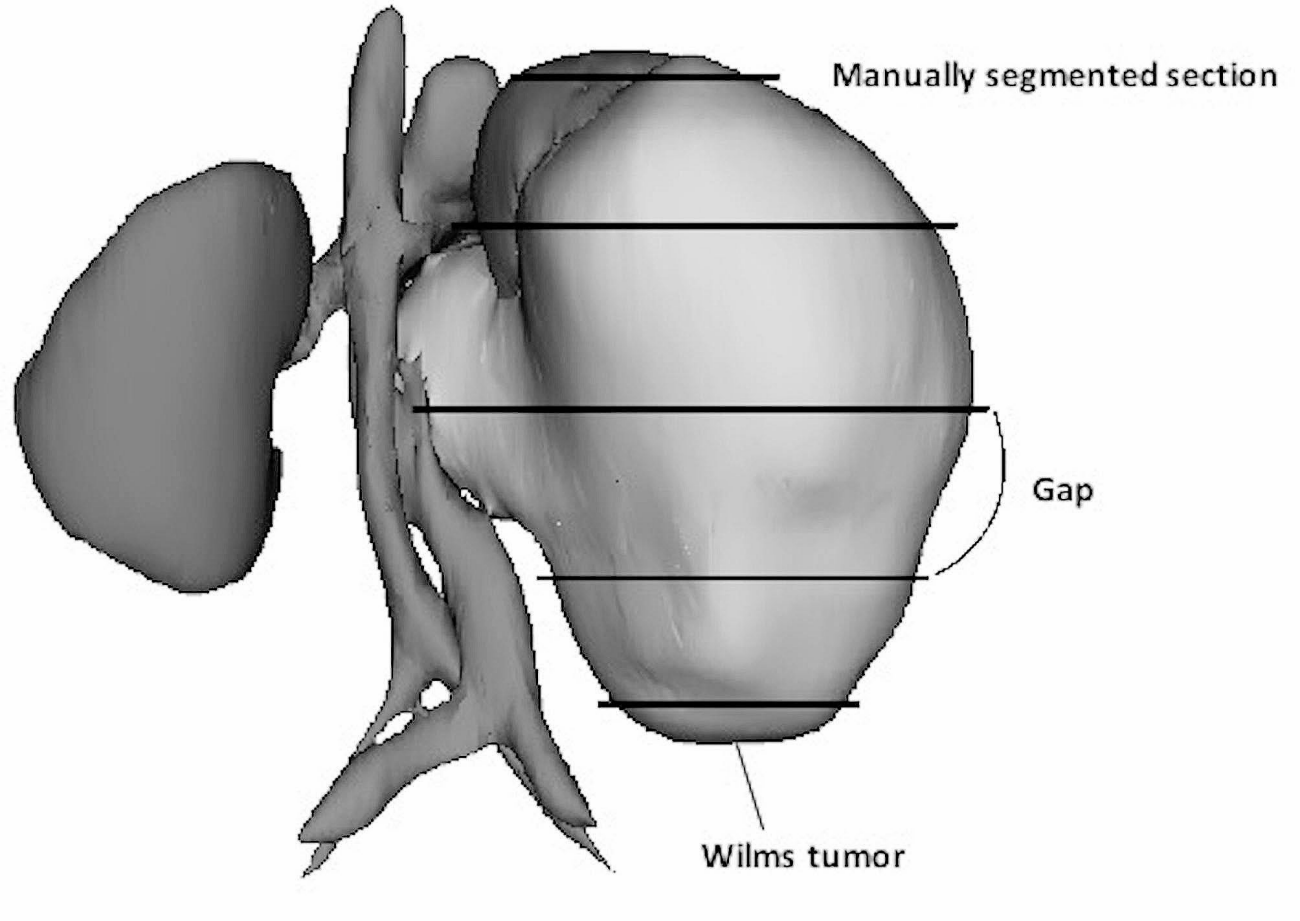
Representation of manually segmented sections and the notion of a gap with the OV^2^ASSION method

The segmentation was then performed with the same CNN U-Net trained according to the OV^2^ASSION (overlearning vector for valid sparse segmentations) training method, described in a previous article [9, 10]. In this method, CNN training is performed with a variable number of patient CT scan sections that have been manually segmented by an expert. The objective of the CNN is then to automatically complete the segmentation of the missing sections. The results obtained are then used to define the minimum number of manually segmented sections so that the CNN completes the segmentation automatically with good accuracy. The notion of “gap”, therefore, defines the interval between two manually segmented sections (Fig. 1). The larger the gap, the greater the interval between two sections and, therefore, the more the need for intervention by an expert is restricted. The time saving for the human expert is estimated depending on the number of sections automatically segmented. Computations have been performed on the supercomputer facilities of the Franche-Comté Computation Mesocenter.

## Evaluation of the results

The segmentations obtained by AI were compared to the manual segmentations performed by an expert using the Dice similarity index according to the following formula:

$${\rm{Dice}}\,{\rm{similarity}}\,{\rm{index}}\,{\rm{ = }}\,\frac{{2\left| {X \cap Y} \right|}}{{\left| {X \cup Y} \right|}}\, = \,\frac{{2A}}{{2A + B + C}}$$

where:

X is the segmentation obtained by AI.

Y is the segmentation performed manually by an expert.

A is the number of common pixels, present in class X and in class Y.

B is the number of pixels present in class Y and absent in class X.

C is the number of pixels present in class X and absent in class Y.

## Results

When segmentations were performed manually by two experts, the inter-individual variability resulted in an average Dice index of 0.95 [0.91–0.97] for Wilms’ tumor (Table 1) and 0.87 [0.69–0.96] for neoplastic kidney (Table 2). The Dice index was greater than 0.90 for all patients concerning the renal tumor and it was less than 0.80 for two patients concerning the neoplastic kidney.

**Table 1:** Dice indices and estimated time saving for the segmentation of Wilms’ tumors with manual segmentation (inter-individual variability), automatic segmentation (CNN U-Net) and semi-automatic segmentation (CNN U-Net trained with the OV^2^ASSION method)

Patient	Inter-individual variability (manual segmentation)	Automatic segmentation CNN U-Net	Semi-automatic segmentation CNN U-Net + OV^2^ASSION		
			Gap 1	Gap 5	Gap 10
1	0.94	0.69	0.99	0.97	0.96
2	0.91	0.43	0.96	0.93	0.92
3	0.95	0.01	0.95	0.86	0.88
4	0.92	0.93	0.98	0.97	0.96
5	0.96	0.88	0.97	0.96	0.95
6	0.96	0.83	0.97	0.96	0.95
7	0.94	0.88	0.98	0.97	0.96
8	0.96	0.84	0.98	0.97	0.96
9	0.95	0.86	0.97	0.96	0.95
10	0.97	0.90	0.98	0.97	0.96
11	0.96	0.61	0.98	0.97	0.96
12	0.95	0.85	0.98	0.96	0.95
13	0.95	0.82	0.97	0.95	0.93
14	0.95	0.19	0.94	0.92	0.90
**Average**	**0.95**	**0.69**	**0.97**	**0.95**	**0.94**
**Estimated time saving**	**0%**	**100%**	**33%**	**71%**	**83%**

**Table 2:** Dice indices and estimated time saving for the segmentation of neoplastic kidneys with manual segmentation (inter-individual variability), automatic segmentation (CNN U-Net) and semi-automatic segmentation (CNN U-Net trained with the OV^2^ASSION method)

Patient	Inter-individual variability (manual segmentation)	Automatic segmentation CNN U-Net	Semi-automatic segmentation CNN U-Net + OV^2^ASSION		
			Gap 1	Gap 5	Gap 10
1	0.83	0.15	0.97	0.95	0.92
2	0.96	0.43	0.97	0.96	0.95
3	0.92	0.41	0.99	0.98	0.97
4	0.88	0.20	0.97	0.95	0.94
5	0.89	0.56	0.93	0.92	0.90
6	0.82	0.17	0.93	0.91	0.88
7	0.92	0.15	0.93	0.86	0.84
8	0.92	0.19	0.97	0.96	0.94
9	0.69	0.05	0.84	0.79	0.45
10	0.79	0.26	0.86	0.82	0.77
11	0.88	0.02	0.94	0.91	0.85
12	0.88	0.23	0.90	0.84	0.82
13	0.88	0.46	0.93	0.91	0.88
14	0.87	0.46	0.99	0.98	0.98
**Average**	**0.87**	**0.27**	**0.94**	**0.91**	**0.86**
**Estimated time saving**	**0%**	**100%**	**33%**	**71%**	**83%**

Of the 14 patients tested, automatic segmentation with the CNN U-Net yielded an average Dice index of 0.69 [0.01–0.93] for Wilms’ tumor segmentation (Table 1) and 0.27 [0.02–0.56] for neoplastic kidney segmentation (Table 2). The Dice index was greater than 0.80 for 9 out of 14 patients regarding renal tumor segmentation. In contrast, the Dice index was less than 0.80 for all patients regarding neoplastic kidney segmentation, with a maximum Dice value of 0.56.

With the OV^2^ASSION drive method, the average Dice index varied depending on the number of manually segmented sections. Logically, the Dice index tends to decrease when the gap increases because it means that the CNN has less established data for training. For the segmentation of the Wilms’ tumors (Table 1), it varied from 0.97 for a gap of 1 (2 out of 3 sections performed manually) to 0.94 for a gap of 10 (1 section out of 6 performed manually). The Dice index was greater than 0.80 for all patients, regardless of the gap used. For neoplastic kidney segmentation (Table 2), the average Dice index ranged from 0.94 for a gap of 1 to 0.86 for a gap of 10. Twelve patients had a Dice index greater than 0.80 for all gaps tested. On the other hand, two patients (patients 9 and 10) had a Dice index below 0.80, with a poor result for patient 9 since the Dice index decreased to less than 0.70 from gap 7 and fell below 0.50 from gap 9.

**Fig. 2:**
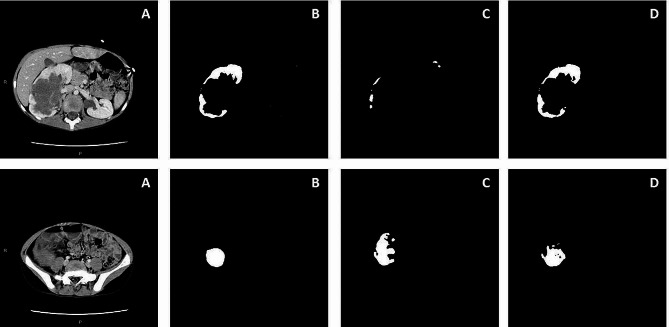
Results of renal (first line) and tumor (second line) segmentations. **Column A**: CT cross-sections. **Column B**: manual segmentations performed by a human expert. **Column C**: automatic segmentations obtained with CNN U-Net. **Column D**: segmentations obtained with CNN U-Net + OV^2^ASSION training method

The Fig. 2 shows an example of renal and tumor segmentations achieved by CNN U-Net with and without OV^2^ASSION method. Thus, the CNN driven by the OV^2^ASSION method obtained identical results to the inter-individual variability for a gap of 7 concerning the Wilms’ tumor and for a gap of 8 concerning the neoplastic kidney. In other words, the segmentation with the AI tool was as precise as the segmentation by an expert when provided with 1 out of 5 manually segmented sections. The time saving for the human expert varied depending on the gap, from 33% for a gap of 1 (1 section out of 3 was automatically segmented) to 83% for a gap of 10 (10 sections out of 12 were automatically segmented).

## Discussion

AI tools are increasingly used for analysis and processing of medical images, allowing different tasks to be performed such as classification, detection, and segmentation [6]. In our study, we used convolutional neural networks for segmentation of Wilms’ tumors in children. Although it is possible to use already developed neural networks, such as U-Net [7] or FCN [11], we found that the results obtained were not satisfactory for segmentation of neoplastic kidneys or Wilms’ tumors in children. Indeed, use of the CNN U-Net on our sample yielded an average Dice index for Wilms’ tumor segmentation (0.69) and a poor index for neoplastic kidney segmentation (0.27). Performing a fully automated segmentation remains a challenge in the field of medical image processing, especially when pathological situations in children are being investigated. Several authors have proposed AI tools to achieve good results with fully automated segmentation of healthy kidneys [12, 13]. However, the situation is complicated when pathological kidneys are examined, whether or not they are malformed [14] or neoplastic [15]. To overcome this problem, a competition was created in adults, called the 2019 Kidney and Kidney Tumor Segmentation Challenge (KiTS19) at the International Conference on Medical Image Computing and Computer Assisted Intervention [16]. This competition had two objectives: (1) to allow a fair and objective comparison of the various methods (since all teams had the same training set and were evaluated by the same metrics on the same test set) and (2) to stimulate research on the challenge of automatic segmentation by making a quantity of established data available for the entire international research community. The results proved to be very good, as the winning team had developed an AI tool capable of automatically segmenting the pathological kidney and the renal tumor with average Dice indices of 0.97 and 0.85, respectively [16]. Other studies have demonstrated good results in renal tumor segmentation in adults using deep learning methods [8, 17, 18]. However, these results are not transferable to Wilms’ tumors in children. Indeed, this tumor type is very different from kidney tumors in adults. They are often much larger, thus compressing the renal parenchyma, which can be very thinned or even fragmented and clearly modify the usual anatomical ratios. In addition, Wilms’ tumor can have a very heterogeneous appearance from one patient to another (regarding its location, size, spatial complexity, intensity, contrast, or relationships with neighboring organs), thus complicating the learning of AI tools.

Another obstacle concerns the quality of medical images, especially CT scans, which is often worse in children than in adults. This can be explained by the use of low-dose acquisition protocols to limit the irradiation of children, by greater movement artifacts during image acquisition, and by a lower amount of fat in children decreasing contrast within the image [3]. It is also possible to use magnetic resonance imaging (MRI). However, the time taken to acquire such images is relatively long, so these examinations may need to be performed under general anesthesia. The progress made in this field with the emergence of more efficient MRI units has reduced the image acquisition time and may hence have reduced this constraint.

The amount of information available (i.e., images segmented and labeled by an expert) is still very limited, as this pathology remains relatively rare. One of the main challenges in medical imaging-based deep learning in kidney diseases is the lack of large, diverse datasets [4]. This is undeniably a major obstacle to the training of AI tools, largely explaining the disappointing results obtained in our study by CNN U-Net.

This is why we have developed a special training method called OV^2^ASSION. This method allows the AI tool to perform learning from a few patient sections manually segmented by an expert. This method is certainly not completely automated, but it allows for a quite significant reduction in the duration of the expert’s intervention. In our study, the CNN managed to automatically complete the segmentation of the neoplastic kidney and Wilms’ tumor with the same accuracy as an expert when it was trained based on one manually segmented section out of 5. If the segmentation time is assumed to be identical for each section, this reduces the intervention time of the expert by 80%.

The original objective has not been fully met as the method presented here is not entirely automated. However, several perspectives are worth further consideration to achieve this. To improve the performance of AI tools, it is essential to increase the amount of information (i.e., source images with their labeled segmentations) for the learning phase. One can then envision creating a database specific to Wilms’ tumor in children that can be used by the entire international scientific community (similar to what was done with the Kidney and Kidney Tumor Segmentation Challenge in adults). It is also possible to artificially increase this amount of information using only computer processes with data augmentation (where the number of images can be artificially increased using rotation, translation, or reversal techniques on the available images).

It would also be worthwhile to optimize the performance of AI tools by providing them with anatomical knowledge [19] so they can reason like an expert when performing manual segmentation. This then raises the question of the organization and prioritization of this knowledge so that it can be read and exploited by computer tools. Several ways can be envisioned to achieve this, such as the use of ontologies (for pathological anatomy) or atlases (for normal anatomy).

The development of a fully automated method remains a complex challenge, especially in regard to the tumor pathology of children. An alternative would, therefore, be to move towards the development of a semi-automated method during which intervention by the expert would be very limited (such as manually positioning the germinating pixel on a section with a single click or marking the boundaries of the tumor by a few reference points on a limited number of sections). Although this method is not fully automated, it would still be readily usable by the operator in daily clinical practice.

Finally, it will be essential to extend these segmentation processes to the arterial vascularization, venous vascularization, and urinary tract since it is paramount that these anatomical elements are also analyzed for decision-making [20]. This will lead to a new challenge to be solved, that of registration, because these structures can only be segmented on images acquired at different times.

## Conclusions

Fully automated segmentation remains a challenge in the field of medical image processing, especially when the tumor pathology in children is assessed. We developed a process for segmentation of Wilms’ tumors and neoplastic kidneys using the CNN U-Net trained according to the OV^2^ASSION method. This technique, which is not fully automated, makes it possible to carry out segmentation of the kidney and its tumor with the same precision as an expert while reducing their intervention time by 80%.

## Data Availability

The data sets generated during and/or analyzed during the current study are not publicly available due to intellectual property/privacy but are available from the corresponding author on reasonable request.

## Abbreviations

AI: Artificial Intelligence
CNN: Convolutional Neural Networks
CT: Computed Tomography
OV^2^ASSION: Overlearning vector for valid sparse segmentations
